# Autologous bone marrow mononuclear cell administration for neurological sequelae after traumatic brain injury: a matched control study

**DOI:** 10.1093/braincomms/fcaf361

**Published:** 2025-09-23

**Authors:** Liem Thanh Nguyen, Giang Thi Huong Ha, Kien Trung Nguyen, Van Thanh Hoang, Quyen Thi Nguyen, Minh Van Pham, Anh Thi Phuong Nguyen, Doan Van Ngo, Huong Thu Le, Chi Van Nguyen

**Affiliations:** Vinmec Research Institute of Stem Cell and Gene Technology, College of Health Sciences, VinUniversity, Hanoi 100000, Vietnam; Department of Rehabilitation Medicine, Ha Noi Medical University, Hanoi 100000, Vietnam; Vinmec Research Institute of Stem Cell and Gene Technology, College of Health Sciences, VinUniversity, Hanoi 100000, Vietnam; Vinmec Research Institute of Stem Cell and Gene Technology, College of Health Sciences, VinUniversity, Hanoi 100000, Vietnam; Vinmec Research Institute of Stem Cell and Gene Technology, College of Health Sciences, VinUniversity, Hanoi 100000, Vietnam; Department of Rehabilitation Medicine, Ha Noi Medical University, Hanoi 100000, Vietnam; Department of Internal Medicine, Ha Noi Rehabilitation Hospital, Hanoi 100000, Vietnam; Department of Hematology & Cell Therapy, Vinmec Times City International Hospital, Hai Ba Trung District, Hanoi 100000, Vietnam; Department of Diagnostic Imaging, Vinmec Times City International Hospital, Hai Ba Trung District, Hanoi 100000, Vietnam; Department of Hematology & Cell Therapy, Vinmec Times City International Hospital, Hai Ba Trung District, Hanoi 100000, Vietnam; Department of General Rehabilitation, National Rehabilitation Hospital, Thanh Hoa 440000, Vietnam

**Keywords:** traumatic brain injury, neurological sequelae, autologous bone marrow mononuclear cells, intrathecal infusion, rehabilitation therapy

## Abstract

Neurological sequelae after traumatic brain injury impair motor and behavioural functions, imposing a heavy burden on patients and society. Traditional treatments show limited efficacy, highlighting the need for advanced therapies. This study evaluated the safety and efficacy of intrathecal administration of bone marrow mononuclear cells for treating these sequelae. A matched control clinical trial was conducted on 50 patients. The intervention group received two intrathecal infusions of bone marrow mononuclear cells, 6 months apart, combined with rehabilitation therapy, while the control group received rehabilitation therapy only. Safety was assessed through adverse events and serious adverse events. Effectiveness was measured via the Functional Independence Measure, Short Form-36 Quality of Life Questionnaire and Glasgow Outcome Scale-Extended. MRI and PET-CT imaging monitored brain changes in the intervention group. No serious adverse events were reported during 12 months of follow-up. Mild adverse events, such as pain at the aspiration site and dizziness, were self-limiting. The intervention group showed significant improvements in motor scores (+4.3 points; *P* = 0.02) and cognitive scores (+1.7 points; *P* = 0.009). Quality of life scores in physical and mental domains improved significantly (*P* < 0.05). MRI revealed structural improvements, while PET-CT indicated enhanced metabolic activity in the brain. Intrathecal administration of bone marrow mononuclear cells is safe and effective in improving motor function and quality of life in patients with neurological sequelae after traumatic brain injury. This therapy is recommended as a complementary treatment alongside rehabilitation therapy.

## Teaser

Nguyen *et al*. report that intrathecal autologous bone marrow mononuclear cell therapy is safe and more effective than standard rehabilitation for improving motor, cognitive function and quality of life in patients with neurological sequelae after traumatic brain injury.

## Graphical Abstract

**Graphical Abstract:**
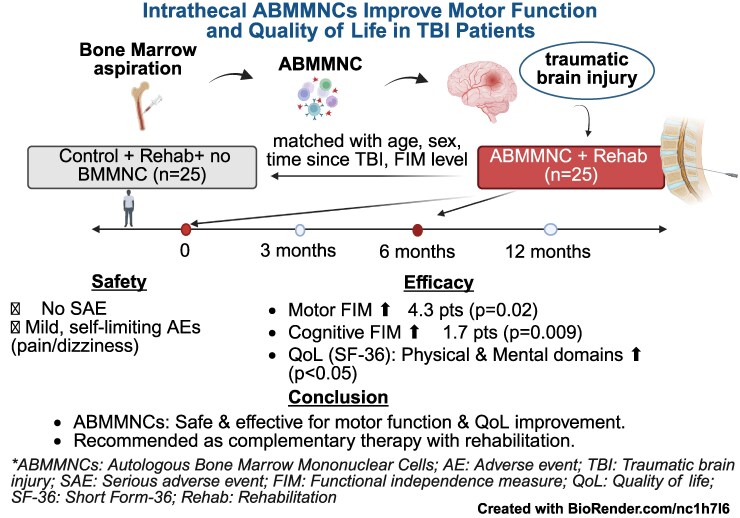

## Introduction

The incidence and prevalence of brain trauma remain high worldwide, with considerable regional variations. In North America, the incidence is 1299 cases per 100 000 people, compared with 1012 cases in Europe and 801 cases in Africa.^1^ Despite advances in surgical and medical treatments, traumatic brain injury (TBI) continues to have a high mortality rate, reaching 20% in severe cases, with an overall rate of 5%.^2^ Among patients admitted to the intensive care unit with moderate-to-severe TBI, only 45% achieve a favourable outcome at 6 months.^3^

TBI often results in severe neuropsychological sequelae. Lloyd *et al*.^4^ (2015) reported that among 8553 children and adolescents aged 0–18 with mild-to-moderate TBI, 50% experienced psychosocial issues, 40% had neuropsychological impairments and 51% faced adverse outcomes. Similarly, a study by Steyerberg *et al*.^3^ revealed that, during long-term follow-up, 67% of severe TBI patients exhibited cognitive impairments, 41% had deficits in executive function, 58% experienced slowed processing speed and 57% had memory deficits. In recent years, cell therapy has emerged as a promising approach for addressing the neuropsychological sequelae of TBI. Cox *et al*.^5^ first demonstrated the safety and feasibility of autologous bone marrow mononuclear cells (ABMMNCs) in 2011 through a study on children with severe TBI, which revealed improved brain structure protection and functional recovery. This was followed by further studies in 2017, which confirmed the safety of ABMMNCs and provided evidence for reduced neuroinflammation and enhanced white matter preservation in adults.^6^ In 2020, Sharma *et al*.^7^ reported significant improvements in neuroplasticity and function in chronic TBI patients receiving repeated ABMMNC infusions. A recent study by Cox *et al*.^8^ (2024) further emphasized the potential of ABMMNCs in TBI therapy, highlighting reductions in intensive care duration and structural brain damage in children. In addition to bone marrow mononuclear cells (BMMNCs), other cell types, including umbilical cord mesenchymal stem cells, bone marrow mesenchymal stem cells (BMMSCs), neural stem cells and cord blood–derived cells, have shown potential for improving neuropsychological outcomes after TBI.^9-11^

While BMMNC therapy has demonstrated safety and efficacy in TBI treatment, controlled studies assessing its impact on managing neurological sequelae are lacking. This matched control study aims to evaluate the safety and efficacy of ABMMNC administration in treating neurological sequelae after TBI.

## Materials and methods

### Ethics approval and consent to participate

The study adhered to the ethical principles outlined in the Declaration of Helsinki and complied with the International Council for Harmonisation of Technical Requirements for Pharmaceuticals for Human Use (ICH) and Good Clinical Practice (GCP) guidelines and local regulations. Informed consent was obtained from all participants. The clinical trial was approved by the Ethics Committee of Vinmec International Hospital (approval number 44/2019/QĐ-VMEC) and the Vietnam Ministry of Health (approval number 2000/QĐ-BYT). This study was registered at ClinicalTrials.gov (ID: NCT05293873). Patients were not required to pay any fees, including those for clinical examinations, laboratory tests, imaging tests, cell harvesting, cell processing, cell infusion, medications, hospital stay and rehabilitation.

### Study design

This is a matched control clinical trial conducted to evaluate the safety and efficacy of ABMMNC therapy in 25 pairs of patients diagnosed with neurological sequelae after TBI.

### Sample size

According to previous research, 40% of severe TBI patients treated with standard hospital protocols exhibited improvements in their Glasgow Outcome Scale-Extended (GOSE) scores at the 1-year follow-up.^12^ In our study, ABMMNC therapy increased the improvement rate to 80%. With the Type I error probability (α) set at 0.05 and a power of 85%, the calculation resulted in a total of 50 patients (25 pairs), which were divided equally into 2 groups.

In the ABMMNC group, 25 patients received 2 intrathecal infusions of ABMMNCs, 6 months apart, combined with rehabilitation therapy and neurotropic medications. In the control group, 25 matched patients received rehabilitation therapy and neurotropic medications but no ABMMNC infusions. Matching was performed one-to-one the basis of sex, age (±5 years), time since injury (±2 months) and Functional Independence Measure (FIM) classification level.

### Patients

#### Inclusion criteria

- Age and sex: Male and female patients aged 20–50 years were included.
- FIM score: ≤80, reflecting complete or partial dependency.
- Post-TBI duration: Between 6 and 36 months.
- Diagnosis of closed TBI.
- Agreement to participate in the study with signed informed consent.

#### Exclusion criteria

- Pre-existing neurological disorders or prior neurological impairment before injury.
- Active infection.
- Organ failure, including renal, hepatic, cardiac or respiratory failure.
- Coagulation disorders or anaemia.
- Open TBI diagnosis.
- Cancer.
- Pregnancy.
- Alcohol dependency or substance abuse.
- Lack of family consent for study participation.
- Severe associated injuries, including spinal cord damage, pelvic injuries or damage to the heart and lungs.

### Study setting

The study was conducted at Vinmec Times City International Hospital from 2020 to 2024.

### Bone marrow mononuclear cell preparation

Bone marrow was harvested from the bilateral anterior superior iliac spines under mask anaesthesia, with a volume of 7 ml per kilogram of body weight, up to a maximum of 350 ml. The collected marrow was processed in an ISO Class 7 cleanroom, where it was layered over Ficoll-Paque PREMIUM (Cytiva, Sweden) (density: 1.077 g/ml) in a 1:1 ratio and centrifuged at 1400 × *g* for 18 min at 4°C to isolate BMMNCs. The BMMNCs were washed twice with phosphate-buffered saline (PBS) and resuspended in 10 ml of autologous plasma for infusion. Quality control of the cell products, including testing for bacteria, mycoplasma and endotoxins, was performed before infusion. The total number of BMMNCs, CD34+ cells and the cell viability were also calculated.

### Intervention

Patients in the BMMNC group were intrathecally administered ABMMNCs via an electric syringe pump for 30 min. After each infusion, patients were sedated and allowed to rest for 2 h before returning to the treatment area. The patients received two infusions with 6-month intervals. Patients in the control group did not receive ABMMNC infusions.

Both groups underwent standard rehabilitation therapy, with 2 weeks of intensive therapy at a hospital or rehabilitation following each infusion, followed by continued home-based rehabilitation exercises. At the hospital, therapy sessions lasted 60 min, 5 days per week, for 2 weeks. These sessions focused on physical therapy (e.g. balance and mobility training), occupational therapy (e.g. cognitive and motor skill development) and speech therapy (e.g. swallowing and communication exercises). After discharge, both the ABMMNC and control groups continued home-based rehabilitation for 6 months after each infusion (ABMMNC group) or an equivalent period (control group), consisting of 60-min sessions, 5 days per week, supported by family members. This ensured equivalent post-discharge rehabilitation intensity between groups. As part of routine care, neurotropic medications were prescribed to both groups.

### Follow-up

All patients were monitored for 12 months post-intervention, with assessments conducted at baseline, 3 months, 6 months and 12 months.

### Outcome measures

#### Safety

Safety was assessed by monitoring adverse events (AEs) and serious AEs (SAEs) throughout the study according to CTCAE version 4.03.^13^

#### Efficiency

Efficacy was assessed via the FIM, the Short Form-36 (SF-36) health survey and the GOSE. In addition, MRI and PET-CT imaging were performed for the ABMMNC group to evaluate functional and structural outcomes.

FIM was used to evaluate patients’ physical, psychological and social functioning. This measure consists of 18 items divided into 2 main categories: motor and cognitive functions. The motor category includes self-care, sphincter control, mobility and locomotion, whereas the cognitive category covers communication and social cognition. Higher FIM scores indicate greater independence and functionality.^14^

The SF-36 was used to evaluate quality of life across eight domains, including physical, role limitations due to physical, pain, general health, vitality, social functioning, role limitations due to emotional and mental health.^15^

The GOSE, an enhanced version of the Glasgow Outcome Scale, classifies recovery after TBI into eight levels, ranging from death (i) to full recovery (viii): (i) death, (ii) vegetative state, (iii) severe disability (complete dependence), (iv) moderate-severe disability (partial dependence), (v) moderate disability (unable to return to work), (vi) moderate disability (partially able to return to work), (vii) good recovery with mild sequelae affecting daily life, and (viii) good recovery with mild sequelae not affecting daily life.^16^

Brain MRI scans were performed on 25 patients in the ABMMNC group at baseline, 6 months and 12 months to evaluate structural and functional changes in the brain. T2-weighted imaging (T2W) was used to detect structural alterations in brain tissue, whereas arterial spin labelling (ASL), a non-invasive and contrast-free technique, was used to measure cerebral perfusion. Improved perfusion indicated better blood flow, vascular recovery and neural regeneration, whereas reduced or unchanged perfusion suggested limited therapeutic effects.

PET-CT imaging was conducted on nine patients in the ABMMNC group at baseline and 12 months to assess changes in brain metabolism. Using fluorodeoxyglucose (FDG) as a tracer, PET-CT was used to measure glucose uptake in critical brain regions such as the frontal and temporal lobes and basal ganglia. Increased glucose metabolism, visualized through colour changes on PET-CT images, reflects improved neurological function and recovery.^17^

#### Patient enrolment

A total of 50 patients with neurological sequelae after TBI were enrolled in the study, including 25 in the ABMMNC group and 25 in the control group. The ABMMNC group received two infusions of ABMMNC, separated by 6 months. In addition to standard medical care, rehabilitation exercises and neurotrophic medications were provided. After the initial infusion, patients underwent follow-up assessments at 3 months, received a second infusion at 6 months and completed final evaluations at 12 months. In this group, 24 patients completed all scheduled procedures, while 1 patient did not return for the final follow-up.

**Figure 1:**
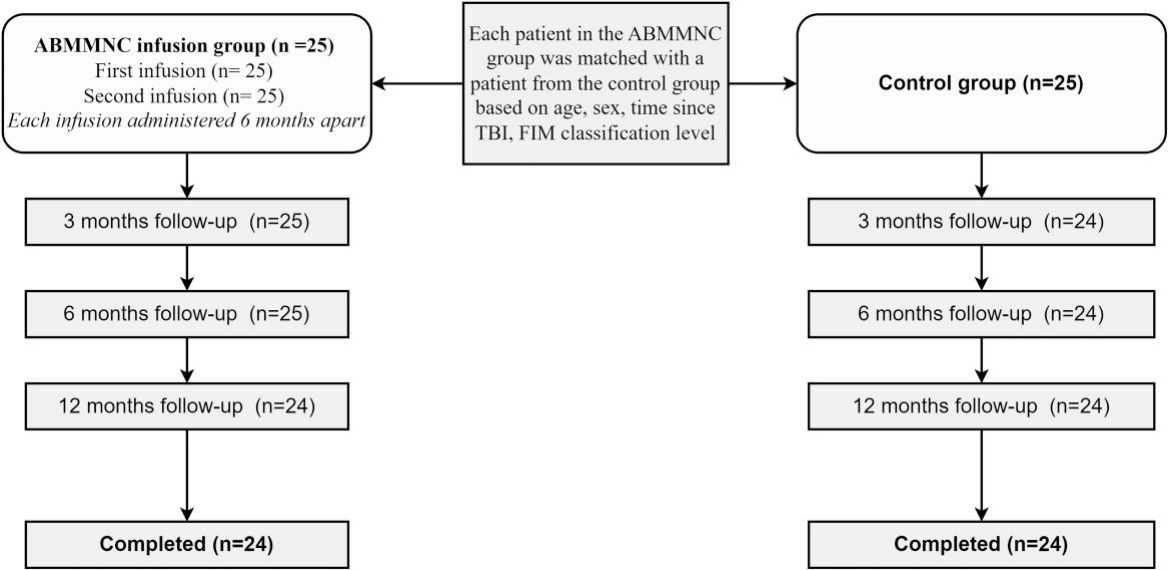
**Flowchart of participant enrolment, allocation and follow-up in the randomized controlled trial.** Fifty patients with neurological sequelae after TBI were allocated to either the ABMMNC therapy group or the control group. Both groups received standard medical care (rehabilitation exercises and neurotrophic medications), with the ABMMNC group additionally receiving two cell infusions at baseline and 6 months. Patients were matched on age, sex, time since TBI and FIM classification level. Follow-up assessments were conducted at 3, 6 and 12 months. One participant in each group was lost to final follow-up.

The control group received only standard medical care comprising rehabilitation exercises and neurotrophic medications, without ABMMNC therapy. The participants in this group were similarly evaluated at 3, 6 and 12 months. Twenty-four patients in the control group completed the study, with 1 patient lost to follow-up at the final assessment (Fig. 1).

### Statistical analysis

Baseline characteristics were summarized via descriptive statistics. Differences in categorical variables, such as sex, age group and GOSE categories, were assessed via the chi-square test. For continuous variables, including the GOSE, FIM and SF-36 scores, one-way ANOVA was used for normally distributed data, whereas the Kruskal–Wallis test was applied for non-normally distributed data.

Before running the mixed-effects model analysis, we used paired *t*-tests (or non-parametric tests for variables with non-normal distribution) to compare the FIM and SF-36 scores at 3, 6 and 12 months with each group’s baseline measurements. This analysis was designed to evaluate how each group changed from baseline over time.

Treatment effects over time were analysed via a mixed-effects model, which incorporated longitudinal data from FIM and SF-36 scores. This model included fixed effects for treatment groups (ABMMNC and control) and random effects to account for individual differences within groups, allowing for a more accurate evaluation of treatment outcomes.

All analyses were performed via STATA software, with statistical significance set at *P* < 0.05.

## Results

Male patients accounted for 84.0% of the patients in the ABMMNC group and 92% of those in the control group (*P* = 0.384; Table 1). The average time since TBI was comparable between the groups (21.6 ± 9.0 versus 21.3 ± 8.3 months, *P* = 0.8966; Table 1). Eighty per cent of patients in the ABMMNC group were classified as having severe disability (GOSE Level 3), compared with 92% in the control group (*P* = 0.144; Table 1). FIM scores were similar in both groups, with 24.0% of patients showing severe limitations (FIM < 40) and 76.0% showing moderate limitations (FIM 40–79; *P* = 1.000; Table 1). The ABMMNC group had a slightly greater mean total FIM score (55.1 ± 20.3 versus 52.3 ± 18.5), but this difference was not statistically significant (*P* = 0.6128; Table 1). Quality of life, measured by the SF-36, was similar between groups, with no significant differences in physical functioning (*P* = 0.4218), emotional well-being (*P* = 0.089) or pain (median 67.5 versus 57.5, *P* = 0.5485; Table 1).

**Table 1:** Baseline characteristics of patients

Characteristics	ABMMNC group (*N* = 25)	Control group (*N* = 25)	*P*-value
Gender, *n* (%)			
Male	21 (84.0%)	23 (92.0%)	0.384
Female	4 (16.0%)	2 (8.0%)	
Age, mean ± SD (years)	32.1 ± 7.7	31.8 ± 7.4	0.8735
Time since TBI, mean ± SD (months)	21.6 ± 9.0	21.3 ± 8.3	0.8966
GOSE, *n* (%)			
2—vegetative state	0 (0.0%)	1 (4.0%)	0.144
3—severe disability (lower level)	20 (80.0%)	23 (92.0%)	
4—severe disability (upper level)	5 (20.0%)	1 (4.0%)	
Categorization by FIM score, *n* (%)			
Severe (FIM <40)	6 (24.0%)	6 (24.0%)	1.000
Moderate (FIM 40–79)	19 (76.0%)	19 (76.0%)	
FIM total score	55.1 ± 20.3	52.3 ± 18.5	0.6128
FIM motor sub-score	37.2 ± 16.9	35.0 ± 14.1	0.632
FIM cognitive sub-score	18.0 ± 5.7	17.3 ± 5.7	0.6739
SF-36, median (min–max)			
Physical functioning	0 (0–45)	0 (0–15)	0.4218
Role limitations due to physical health	0 (0–0)	0 (0–0)	0.3173
Role limitations due to emotional	0 (0–100)	0 (0–100)	0.302
Energy/fatigue	50 (0–70)	40 (15–85)	0.2376
Emotional well-being	52 (4–80)	60 (24–76)	0.089
Social functioning	25 (0–100)	37.5 (0–62.5)	0.1558
Pain	67.5 (32.5–100)	57.5 (10–100)	0.5485
General health	35 (25–65)	35 (20–50)	0.5022

SF-36, Short Form-36.

**Figure 2:**
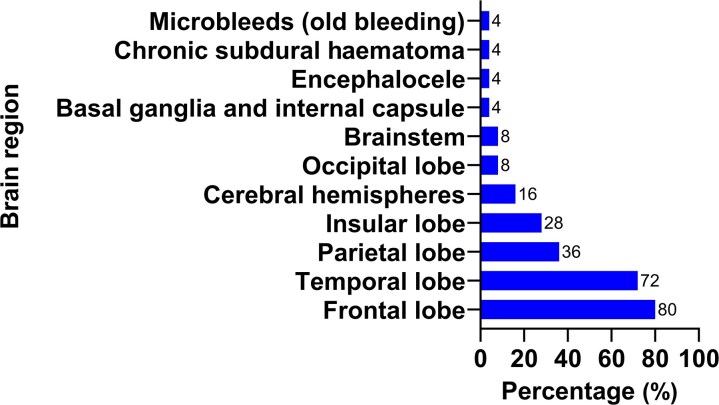
**Distribution of brain lesion locations on baseline MRI in the ABMMNC group.** Bar chart shows the percentage of patients (*N* = 25) with lesions in each brain region at baseline. The frontal lobe was the most frequently affected (80%), followed by the temporal lobe (72%), parietal lobe (36%), insular lobe (28%), cerebral hemispheres (16%), occipital lobe (8%) and brainstem (8%). Rare findings (4%) included lesions in the basal ganglia and internal capsule, encephalocele, chronic subdural haematoma and microbleeds (old bleeding). Statistical analysis: Data are presented as descriptive frequencies (%). No statistical test was applied.

Baseline MRI scans revealed that the frontal lobe was most often affected, with lesions detected in 80% of patients, followed by the temporal lobe (72%). The parietal and insular lobes were involved in 36 and 28% of patients, respectively, whereas the cerebral hemisphere (16%), occipital lobe (8%) and brainstem (8%) were less frequently affected. The rare findings, each found in 4% of patients, included lesions in the basal ganglia/internal capsule, brain herniation, chronic subdural haematoma and microbleeds (old haemorrhages). The distribution of these lesions is summarized in Fig. 2.

### Characteristics of the autologous bone marrow mononuclear cell

The ABMMNC infusions met quality standards. The first infusion had average counts of 19.0 million mononuclear cells (MNCs), 0.4 million CD34+ cells and 0.004 million mesenchymal stem cells (MSCs) per kilogram, with 98.4% cell viability. The second infusion had 16.2 million MNCs, 0.4 million CD34+ cells and 0.003 million MSCs per kilogram, with 98.0% cell viability. All products were sterile, mycoplasma tests were negative and endotoxin levels were below 0.05 endotoxin units (EU)/ml (Supplementary Table 1).

### Safety

In the ABMMNC group, a total of 38 AEs were recorded during the study, including 9 related directly to the infusion. These included mild infusion-site pain (three cases), minor bleeding, swelling at the bone marrow aspiration site, swelling with discomfort in the right forearm due to venous extravasation, hand numbness, nausea and dizziness (one case each). All were mild, self-resolving and required no intervention.

Two SAEs were reported. In the first case, a participant who had a history of post-traumatic seizures developed myoclonic jerking, stridor and a temporary drop in oxygen saturation (SpO_2_) to 92–93% after the second infusion. These symptoms were quickly managed with oxygen therapy, ventolin via a mask and depakine (200 mg, four tablets daily in two doses), resulting in an improvement in SpO_2_ to 97–98% and full recovery of the patient.

The second SAE involved a 21-year-old male with severe disability due to TBI sequelae, who had a baseline FIM score of 20 and a GOSE score of 3, indicating severe dependency. The first and second cell infusions were performed without any AEs/SAEs. At 6 months post-infusion, his GOSE score was 3 and his FIM score was 20. Four months after the second infusion, the patient was admitted to a provincial hospital for severe pneumonia due to respiratory failure. Despite intensive care, his condition did not improve, leading to his discharge at his family’s request.

These two SAEs were not directly associated with the intervention after careful analysis.

In the control group, over a 12-month follow-up period, nine patients experienced AEs. Two patients experienced seizures—one experienced recurrent seizures every 15–17 days to once per month, with each episode lasting 30–60 min, while the other experienced a focal seizure in the paralyzed arm accompanied by severe headache. Additionally, two patients reported headaches (one triggered by weather changes and one with severe headache), one patient developed chickenpox, two patients presented respiratory symptoms (nasal congestion and cough) managed with amoxicillin, one patient reported wound pain associated with weather changes and one patient experienced muscle strain/soft tissue injury in the thigh following strenuous exercise, which stabilized after 1 month of treatment.

Details of all AEs are provided in Supplementary Table 2.

### Efficiency

#### Improvements in the Functional Independence Measure scores

In the ABMMNC group, the total FIM score significantly increased from 55.1 ± 20.3 at baseline to 63.4 ± 22.5 at 3 months (*P* < 0.0001), 66.2 ± 24.1 at 6 months (*P* < 0.0001) and 74.3 ± 24.1 at 12 months (*P* < 0.0001). Motor scores increased from 37.2 ± 16.9 at baseline to 44.6 ± 18.3 at 3 months, 46.4 ± 19.4 at 6 months and 51.3 ± 20.1 at 12 months (all *P* < 0.0001). The cognitive scores improved from 18.0 ± 5.7 at baseline to 18.9 ± 5.3 at 3 months (*P* = 0.0322), 19.8 ± 5.9 at 6 months (*P* = 0.001) and 23.0 ± 5.2 at 12 months (*P* < 0.0001) (see Supplementary Figs. 4–6).

In the control group, the total FIM score also increased from 51.2 ± 18.1 at baseline to 54.5 ± 19.4 at 3 months (*P* = 0.0008), 58.7 ± 21.5 at 6 months (*P* = 0.0001) and 62.7 ± 23.8 at 12 months (*P* = 0.0001). Motor scores improved from 34.0 ± 13.4 at baseline to 36.6 ± 14.9 at 3 months (*P* = 0.0038), 39.9 ± 17.1 at 6 months (*P* = 0.0005) and 42.6 ± 18.8 at 12 months (*P* = 0.0002). The cognitive scores increased from 17.2 ± 5.8 at baseline to 17.9 ± 5.6 at 3 months (*P* = 0.1343), 18.8 ± 5.5 at 6 months (*P* = 0.0072) and 20.0 ± 6.0 at 12 months (*P* = 0.0006) (see Supplementary Figs. 4–6).

The mixed-effects model analysis revealed that both groups’ FIM scores improved over time, with the ABMMNC group showing greater gains at 3 and 12 months. At 3 months, the ABMMNC group gained an additional 5.1 points [95% confidence interval (CI): (1.0, 9.1), *P* = 0.015] compared with the control group. At 6 months, the improvement score was 3.5 points [95% CI: (−0.5, 7.6), *P* = 0.089], and at 12 months, it was 6.0 points [95% CI: (1.9, 10.1), *P* = 0.004] (Table 2).

**Table 2:** Comparison of FIM score changes over time between the ABMMNC group and the control group via a mixed-effects model

Model parameter	Estimate ± SE	95% CI	*P*
Intercept (control group)	51.2 ± 4.5	(42.4; 60.0)	0.533
Baseline comparison	3.9 ± 6.3	(−8.3; 16.2)	
Time point × treatment group			
3 months × ABMMNC group	5.1 ± 2.1	(1.0; 9.1)	0.015
6 months × ABMMNC group	3.5 ± 2.1	(−0.5; 7.6)	0.089
12 months × ABMMNC group	6.0 ± 2.1	(1.9; 10.1)	0.004

‘Intercept (control group)’ represents the baseline FIM score of the control group. ‘Baseline comparison’ indicates the estimated difference between ABMMNC group and the control group at baseline. ‘Time point × treatment group’ represents the change in FIM scores at each time point for the ABMMNC group compared with the control group.

The mixed-effects model analysis indicated that both groups showed improvements in motor FIM scores over time, with the ABMMNC group showing greater increases. At 3 months, the ABMMNC group improved by an additional 4.8 ± 1.8 points compared with the control group [95% CI: (1.3, 8.4); *P* = 0.008]. By 6 months, this increase was 3.3 ± 1.8 points [95% CI: (−0.2, 6.9); *P* = 0.067], and at 12 months, it was 4.3 ± 1.8 points [95% CI: (0.7, 7.8); *P* = 0.02] (Supplementary Table 3).

The mixed-effects model analysis revealed that both groups showed improvements in their cognitive FIM scores over time, but the ABMMNC group exhibited a greater improvement at 12 months. At 3 months, the ABMMNC group had an increase of 0.3 ± 0.6 points compared with the control group [95% CI: (−1.0, 1.5); *P* = 0.7]. At 6 months, the improvement score was 0.2 ± 0.6 points [95% CI: (−1.1, 1.5); *P* = 0.742]. By 12 months, the ABMMNC group showed a significant improvement of 1.7 ± 0.6 points [95% CI: (0.4, 3.0); *P* = 0.009] (Supplementary Table 4).

#### SF-36 Health Survey

In the ABMMNC group, the SF-36 score increased over time (Supplementary Table 5). Physical function increased from a median of 0 (0–10) at baseline to 25 (5–55.7) at 12 months (*P* < 0.001), pain increased from 67.5 (45–100) to 100 (100–100) (*P* < 0.001) and general health increased from 35 (35–45) to 60 (55–72.5) (*P* < 0.001). Similarly, mental domain scores, including vitality, social functioning, role limitations due to emotional and mental health, statistically increased (*P* < 0.001 for most comparisons; Supplementary Table 6). In the control group, changes were modest, with physical function increasing from 0 (0–5) to 15 (0–25) (*P* = 0.024) and pain increasing from 57.5 (47.5–100) to 80 (55–100) (*P* = 0.006).

The mixed-effects model results show that both groups improved in term of physical functioning and role limitations due to physical problems over time. However, the ABMMNC group showed greater improvements at several points. Compared with those in the control group, physical function in the ABMMNC group increased by 13.4 points [95% CI: (2.3, 24.4); *P* = 0.018] at 3 months, 12.3 points [95% CI: (1.2, 23.4); *P* = 0.03] at 6 months and 13.3 points [95% CI: (2.1, 24.4); *P* = 0.02] at 12 months. For role limitations due to physical problems, the ABMMNC group improved by 6.2 points [95% CI: (−2.4, 14.8); *P* = 0.159] at 3 months, 4.2 points [95% CI: (−4.4, 12.8); *P* = 0.341] at 6 months and 13.5 points [95% CI: (4.9, 22.2); *P* = 0.002] at 12 months (Table 3).

**Table 3:** Comparison of changes in SF-36 scores for physical and role limitations due to physical domains over time between the groups via a mixed-effects model

Model parameter	Physical			Role limitations due to physical		
	Estimate ± SE	95% CI	*P*	Estimate ± SE	95% CI	*P*
Intercept (control group)	51.3 ± 17.3	(17.4; 85.2)	0.977	3.1 ± 7.6	(−11.9; 18.0)	0.148
Baseline comparison	0.2 ± 5.4	(−10.4; 10.7)		4.6 ± 3.2	(−1.7; 10.9)	
Time point × treatment group						
3 months × ABMMNC	13.4 ± 5.6	(2.3; 24.4)	0.018	6.2 ± 4.4	(−2.4; 14.8)	0.159
6 months × ABMMNC	12.3 ± 5.6	(1.2; 23.4)	0.03	4.2 ± 4.4	(−4.4; 12.8)	0.341
12 months × ABMMNC	13.3 ± 5.7	(2.1; 24.4)	0.018	13.5 ± 4.4	(4.9; 22.2)	0.002

‘Intercept (control group)’ represents the baseline SF36 score of the control group. ‘Baseline comparison’ indicates the estimated difference between ABMMNC group and the control group at baseline. ‘Time point × treatment group’ represents the change in SF-36 scores at each time point for the ABMMNC group compared with the control group.

In the ABMMNC group, pain scores increased by 8.6 points at both the 3-month mark [95% CI: (−2.8, 20.1); *P* = 0.138] and the 6-month mark [95% CI: (−2.8, 20.0); *P* = 0.141]. By 12 months, however, there was an improvement in pain scores of 11.5 points [95% CI: (0.0, 23.0); *P* = 0.049]. The general health parameters steadily increased over time, with increases of 5.1 points at 3 months [95% CI: (−0.5, 10.8); *P* = 0.077], 8.0 points at 6 months [95% CI: (2.3, 13.6); *P* = 0.006] and 10.8 points at 12 months [95% CI: (5.1, 16.5); *P* < 0.001] (Table 4).

**Table 4:** Comparison of changes in SF-36 scores for pain and general health domains over time between the groups via a mixed-effects model

Model parameter	Pain			General health		
	Estimate ± SE	95% CI	*P*	Estimate ± SE	95% CI	*P*
Intercept (control group)	85.2 ± 22.9	(40.4; 130.0)	0.131	17.8 ± 8.7	(0.8; 34.8)	0.48
Baseline comparison	9.8 ± 6.5	(−2.9; 22.6)		1.9 ± 2.7	(−3.4; 7.2)	
Time point × treatment group						
3 months × ABMMNC	8.6 ± 5.8	(−2.8; 20.1)	0.138	5.1 ± 2.9	(−0.5; 10.8)	0.077
6 months × ABMMNC	8.6 ± 5.8	(−2.8; 20.0)	0.141	8.0 ± 2.9	(2.3; 13.6)	0.006
12 months × ABMMNC	11.5 ± 5.9	(0.0; 23.0)	0.049	10.8 ± 2.9	(5.1; 16.5)	<0.001

‘Intercept (control group)’ represents the baseline SF36 score of the control group. ‘Baseline comparison’ indicates the estimated difference between ABMMNC group and the control group at baseline. ‘Time point × treatment group’ represents the change in SF-36 scores at each time point for the ABMMNC group compared with the control group.

In the ABMMNC group, vitality scores increased over time, with gains of 10.5 points at 3 months [95% CI: (1.8, 19.3); *P* = 0.019], 13.0 points at 6 months [95% CI: (4.2, 21.7); *P* = 0.004] and 14.6 points at 12 months [95% CI: (5.8, 23.3); *P* = 0.001). The social functioning scores also increased, with increases of 5.1 points at 3 months [95% CI: (−8.6, 18.8); *P* = 0.466], 16.8 points at 6 months [95% CI: (3.1, 30.5); *P* = 0.016] and 20.2 points at 12 months [95% CI: (6.5, 34.0); *P* = 0.004] (Table 5).

**Table 5:** Comparison of changes in the SF-36 scores for the vitality and social functioning domains over time between the groups via a mixed-effects model

Model parameter	Vitality			Social functioning		
	Estimate ± SE	95% CI	*P*	Estimate ± SE	95% CI	*P*
Intercept (control group)	40.3 ± 13.5	(13.9; 66.8)	0.893	22.2 ± 22.2	(−21.3; 65.6)	0.606
Baseline comparison	0.6 ± 4.4	(−8.0; 9.2)		3.7 ± 7.2	(−10.4; 17.9)	
Time point × treatment group						
3 months × ABMMNC	10.5 ± 4.5	(1.8; 19.3)	0.019	5.1 ± 7.0	(−8.6; 18.8)	0.466
6 months × ABMMNC	13.0 ± 4.5	(4.2; 21.7)	0.004	16.8 ± 7.0	(3.1; 30.5)	0.016
12 months × ABMMNC	14.6 ± 4.5	(5.8; 23.3)	0.001	20.2 ± 7.0	(6.5; 34.0)	0.004

‘Intercept (control group)’ represents the baseline SF36 score of the control group. ‘Baseline comparison’ indicates the estimated difference between ABMMNC group and the control group at baseline. ‘Time point × treatment group’ represents the change in SF-36 scores at each time point for the ABMMNC group compared with the control group.

For role limitations caused by emotional problems, the ABMMNC group initially showed minor changes: a decrease of 5.9 points at 3 months [95% CI: (−30.5, 18.6); *P* = 0.636] and an increase of 0.9 points at 6 months [95% CI: (−23.7, 25.5); *P* = 0.943]. However, by 12 months, there was a significant improvement of 27.7 points [95% CI: (3.0, 52.4); *P* = 0.028]. In term of mental health, the ABMMNC group improved by 9.9 points at 3 months [95% CI: (2.3, 17.5); *P* = 0.011], 16.6 points at 6 months [95% CI: (9.0, 24.3); *P* < 0.001] and 19.4 points at 12 months [95% CI: (11.7, 27.0); *P* < 0.001] (Table 6).

**Table 6:** Comparison of changes in SF-36 scores for domain role limitations due to emotional and mental health over time between the groups via a mixed-effects model

Model parameter	Role limitations due to emotional			Mental health		
	Estimate ± SE	95% CI	*P*	Estimate ± SE	95% CI	*P*
Intercept (control group)	24.5 ± 33.7	(−41.6; 90.6)	0.623	73.9 ± 13.3	(47.9; 99.9)	0.202
Baseline comparison	−5.4 ± 10.9	(−26.8; 16.1)		−5.5 ± 4.3	(−14.0; 3.0)	
Time point × treatment group						
3 months × ABMMNC	−5.9 ± 12.5	(−30.5; 18.6)	0.636	9.9 ± 3.9	(2.3; 17.5)	0.011
6 months × ABMMNC	0.9 ± 12.5	(−23.7; 25.5)	0.924	16.6 ± 3.9	(9.0; 24.3)	<0.001
12 months × ABMMNC	27.7 ± 12.6	(3.0; 52.4)	0.028	19.4 ± 3.9	(11.7; 27.0)	<0.001

‘Intercept (control group)’ represents the baseline SF36 score of the control group. ‘Baseline comparison’ indicates the estimated difference between ABMMNC group and the control group at baseline. ‘Time point × treatment group’ represents the change in SF-36 scores at each time point for the ABMMNC group compared with the control group.

### Improvement in Glasgow Outcome Scale-Extended

Improvement rates in GOSE scores were assessed at 3, 6 and 12 months after treatment. At 3 months, no improvements were observed in the ABMMNC group, whereas the control group showed an improvement rate of 8.3% (*P* = 0.141). At 6 months, the improvement rate in the ABMMNC group was 16%, whereas it was 12.5% in the control group (*P* = 0.726). By 12 months, the ABMMNC group had a greater improvement rate of 26.1 versus 20.8% in the control group (*P* = 0.671) (Supplementary Table 7).

#### MRI of the brain

Brain MRI scans were conducted on all 25 patients in the ABMMNC group at baseline, 6 months and 12 months to evaluate structural changes in brain tissue and blood perfusion in affected regions.

##### T2-Weighted imaging

At 6 months, there were no changes in the size or signal intensity of the lesions observed on T2W imaging in any of the 25 patients, indicating that the lesions remained stable. No new lesions, degeneration or secondary complications were identified. At 12 months, among the 24 patients who completed follow-up, 1 patient (4.2%) displayed a reduction in the size of a pre-existing lesion, with T2W signals indicating reduced oedema. In the remaining 23 patients (95.8%), the lesions maintained their original size without significant changes.

##### Arterial spin labelling

ASL imaging was used to evaluate cerebral blood flow in damaged regions. At 6 months, 4 of 25 patients (16.0%) demonstrated increased perfusion, primarily in the frontal and temporal lobes. After 12 months, the increase in these patients was no longer observed. Four of 24 patients (16.7%) who did not show any improvement in perfusion at 6 months actually showed enhanced perfusion at the 12-month mark.

#### Brain PET/CT

In this study, nine patients from the ABMMNC group underwent brain PET/CT scans via the tracer (^18^F)-FDG before and after the intervention. Among the nine patients, six exhibited increased FDG metabolism in specific brain regions, two exhibited decreased metabolism and one showed no changes.

Patient PID (Patient Identification) 05 exhibited increased FDG metabolism in the left occipital and temporal lobes. Patients PID 02, PID 06, PID 09, PID 10 and PID 11 presented increased metabolism in the basal ganglia, thalamus and cerebellum, particularly in the cerebellar cortex, whereas no significant changes were observed in the frontal, temporal, parietal or occipital lobes (Supplementary Fig. 7). Patient PID 03 demonstrated reduced metabolism in the brainstem alongside increased metabolism in the basal ganglia, thalamus and cortical areas. Patient PID 04 presented with reduced metabolism in the right frontal, right parietal and right temporal regions, as well as in the corpus callosum and left medial frontal gyrus. Patient PID 08 exhibited no changes in metabolism.

## Discussion

This is the first matched control clinical trial to assess the effectiveness of ABMMNCs in treating neurological sequelae after TBI. At baseline, both groups were comparable with no significant differences in key factors such as age, sex, time from injury to intervention, GOSE score, FIM score or quality of life.

Our study confirmed the safety of intrathecal ABMMNC infusions. A total of nine AEs were reported, all of which were mild and resolved without intervention. Six months post-infusion, laboratory parameters, including alanine aminotransferase (ALT), aspartate aminotransferase (AST), uraemia, creatinaemia levels, prothrombin time, thrombin time and activated partial thromboplastin time (APTT), were assessed within normal ranges and showed no significant changes compared with the baseline values (Supplementary Figs. 1–3). Two SAEs were not directly associated with cell therapy.

In contrast, in the control group (*n* = 25) during 12 months of follow-up, we observed nine patients with AEs typical of chronic TBI sequelae: two patients reported seizures, two reported headaches, one experienced chickenpox that required hospitalization, two experienced respiratory infections, one reported wound pain and one experienced a muscle strain/soft tissue injury.

The safety profile of ABMMNC harvesting and administration has been supported by multiple studies. Cox *et al*.^5^ (2011), in a study of children aged 5–14 years with severe TBI, reported no infusion-related toxicity or haemodynamic instability during bone marrow harvesting or infusion. In their study, all 10 children survived, and no adverse impacts on hepatic, renal or pulmonary functions were detected. Tian *et al*.^18^ (2013) reported the absence of severe complications in 97 patients treated with ABMMNCs via lumbar puncture. Similarly, Wang *et al*.^9^ (2013) highlighted the safety of umbilical cord mesenchymal stem cell therapy in 20 patients, with only mild AEs, such as dizziness and headache, observed across four rounds of intrathecal administration per patient. Sharma *et al*.^7^ (2020) reported no deaths or SAEs among 50 patients receiving intrathecal ABMMNCs, with only mild, transient AEs observed, such as fever and headaches.^7^ Cox *et al*. (2024) further supported these findings, demonstrating that ABMMNC infusions were not associated with severe AEs in paediatric patients.^8^ Our findings, combined with these studies, reinforce the safety of intrathecal ABMMNC administration for patients with neurological sequelae after TBI.

Our findings support the effectiveness of ABMMNC therapy in improving functional outcomes for patients with significant motor and cognitive deficits. At 12 months, the ABMMNC group presented an average increase of 19.2 points in total FIM scores from baseline, whereas the control group presented a minor improvement of 11.5 points (*P* < 0.001). Furthermore, mixed-effects model analysis revealed greater improvements in the ABMMNC group showed greater improvements than in the control group at 3, 6 and 12 months, with *P*-values of 0.015, 0.089 and 0.04, respectively.

Motor FIM scores increased by an average of 14.1 points in the ABMMNC group and 8.6 points in the control group from baseline (see Supplementary Fig. 5). This greater improvement in the ABMMNC group indicated enhanced mobility, self-care and independence in daily activities. Using a mixed-effects model, we found that the improvement in motor scores in the ABMMNC group was greater than that in the control group at 3, 6 and 12 months, with *P*-values of 0.008, 0.089 and 0.02, respectively.

The cognitive FIM scores increased by an average of 5.0 points in the cell therapy group and 2.8 points in the control group from baseline (see Supplementary Fig. 6). Mixed-effects model analysis revealed that by 12 months, the cell therapy group experienced an additional gain of 1.4 points (*P* = 0.009), reflecting improved social cognition and communication skills.

The observed improvements in FIM scores closely mirrored enhancements in quality of life, as reflected in the SF-36 assessments. The cell therapy group exhibited significant and sustained increases across multiple domains of the SF-36. Specifically, improvements in physical functioning were evident at all time points, with statistically significant differences compared with those of the control group at 3 months (+13.4 points, *P* = 0.018), 6 months (+12.3 points, *P* = 0.03) and 12 months (+13.3 points, *P* = 0.018). Similar trends were noted in role limitations due to physical problems, with the ABMMNC group showing significant gains at 12 months (+13.5 points, *P* = 0.002). Notably, by the 12-month mark, there were substantial improvements in general health (+10.8 points, *P* < 0.001) and mental health (+19.4 points, *P* < 0.001). Social functioning and vitality also demonstrated statistically significant enhancements over time. At 12 months, the ABMMNC group achieved a 20.2 point gain in social functioning (*P* = 0.004) and a 14.6 point increase in vitality (*P* = 0.001), highlighting the broader psychosocial benefits of the intervention. These findings show that cell therapy can restore physical function and improve mental and emotional health, helping to accomplish a full recovery.

The improvement rates on the GOSE scale were consistently greater in the ABMMNC group than in the control group, reaching 26.1% at 12 months versus 20.8% in the control group. Although these differences did not reach statistical significance (*P* = 0.671), the trends suggest a meaningful clinical impact.

The reason for the difference between improvements in FIM/SF-36 scores and the lack of change in GOSE scores is mainly due to how these tools measure outcomes. FIM and SF-36 are detailed and can pick up small improvements in everyday function and quality of life, while GOSE uses broad categories and only shows change when patients make big jumps in recovery. Since most patients in our study had severe disability at the start (GOSE ≤4), even clear improvements in daily function may not be enough to move them to a higher GOSE category. Also, our study may not have had enough patients to reliably detect GOSE changes, and GOSE improvements often take more than 12 months to appear, especially when using structured interviews as recommended by Wilson *et al*.^19^ (1998). This explains why improvements in FIM/SF-36 were seen but not in GOSE. This finding is consistent with Sharma *et al*.’s^7^ observation that GOSE requires extended follow-up to capture meaningful transitions in severe TBI populations.

ABMMNC therapy promotes neurological recovery after TBI through multiple complementary mechanisms. First, ABMMNCs secrete angiogenic growth factors including vascular endothelial growth factor (*VEGF*) and *HGF*, stimulating cerebral angiogenesis and perfusion. This is supported by increased ASL-MRI perfusion in 16% of patients in our study and corroborates pre-clinical models of *VEGF*-mediated vascular repair.^20,21^ Second, mitochondrial transfer from infused cells enhances neuronal bioenergetics, demonstrated by improved glucose uptake on PET-CT in 67% (6/9) of patients in our study and aligned with studies confirming mitochondrial fusion supports neurovascular function.^22^ Third, ABMMNC-derived extracellular vesicles modulate neuroinflammation by polarizing microglia from pro-inflammatory (M1) to reparative (M2) phenotypes, reducing reducing tumor necrosis factor (TNF)-α levels in pre-clinical models.^23^ This anti-inflammatory effect likely contributes to significant cognitive improvements in our cohort (+1.7 FIM points; *P* = 0.009). Finally, there is *in vitro* evidence suggesting that ABMMNCs may differentiate into neural lineages, which could potentially contribute to tissue repair.^24^ These mechanisms rationalize the significant motor (+4.3 FIM points; *P* = 0.02) and cognitive recovery observed in our study population.

Our findings are consistent with other studies that used cell therapy for brain trauma. Sharma *et al*.^7^ (2020) reported that 92% of patients with chronic TBI showed significant improvements after ABMMNC therapy. These improvements included better voluntary control, posture, cognition and daily activities, with FIM scores increasing in 60% of the participants.

Similarly, Cox *et al*.^8^ (2024) investigated intravenous ABMMNC in children with severe TBI. They reported that the treatment reduced ICU stays and lowered the need for intensive therapeutic measures, while also preserving white matter structure and enhancing corpus callosum connectivity.

Furthermore, Tian *et al*.^18^ administered BMMSCs intrathecally to patients in a persistent vegetative state with motor dysfunction. They reported significant improvements in both consciousness and motor skills, especially among younger patients and those receiving early treatment.^18^

In 2024, Okonkwo *et al*.^11^ conducted a randomized, double-blind, surgical sham-controlled trial evaluating the safety and efficacy of intracranial implantation of allogeneic mesenchymal stromal cells (SB623) in patients with chronic motor deficits following TBI. The trial included 63 participants with moderate-to-severe chronic TBI and motor impairments persisting for at least 12 months. At 24 weeks, patients in the SB623-treated groups demonstrated a significant improvement in motor function, with a mean Fugl-Meyer Motor Scale (FMMS) increase of +8.3 compared with +2.3 in the control group (*P* = 0.04).

Many studies have been conducted in animal models of TBI to understand the mechanism of cell administration. Bedi *et al*.^23^ reported that ABMMNCs increase the apoptosis of activated microglia/macrophages in the hippocampus via cleaved caspase 3, without affecting neuronal apoptosis, ultimately improving cognitive function. ABMMNCs also promote nerve function recovery in rats with TBI by increasing the expression of brain-derived neurotrophic factor (*BDNF*) mRNA and inhibiting the apoptosis of cortical neurons.^25^ Endothelial progenitor cells, a component of BMMNCs, reduce β-amyloid precursor protein build-up in the corpus callosum after fluid percussion injury, improved white matter integrity, and decrease capillary breakdown in adult Sprague–Dawley rats with TBI.^26^ BMMSCs, a stem cell component of ABMMNCs, have been shown to migrate to the brain, kidney, liver, heart, lung and spleen in TBI rats. They express both the neuronal marker NeuN and the astrocytic marker glial fibrillary acidic protein (GFAP), and they significantly reduce motor and neurological deficits, as measured by the Rotarod test.^27^ BMMSCs also increased the expression of *VEGF* and *BDNF*, reduced cortical neuronal death, migrated to injured areas where they differentiated into neurons or astrocytes, and mitigated synaptic protein loss in a TBI rat model.^28^ Treatment with MSCs enhances progenitor cell proliferation in the sub-ventricular zone and boundary zone in Wistar rats with TBI.^29^

Despite these promising results, some limitations in our study should be acknowledged. The 12-month follow-up may not fully capture the long-term durability of the effects, warranting a longer follow-up in future studies.

A key limitation of this study is the absence of randomization in group assignment. Instead of randomly allocating participants, we matched individuals in each group based on important baseline characteristics, specifically sex, age (±5 years), time since injury (±2 months) and FIM classification level. While this matching helps reduce confounding, it does not fully eliminate the risk that the groups may have differed in ways that could influence the results. Additionally, the assessors were not blinded to group allocation. As a result, their knowledge of which participants belonged to each group could have introduced bias into the evaluation of outcomes.

## Conclusion

Intrathecal ABMMNC therapy has been proven to be a safe intervention and could improve motor and cognitive functions, as well as overall quality of life in patients suffering from neurological sequelae after TBI. The results of this study clearly show that patients treated with ABMMNC experienced greater improvements than did those in the control group.

## Supplementary Material

fcaf361_Supplementary_Data

## Data Availability

The data that support the findings of this study are available on request from the corresponding author. The codes generated and used during the current study are available in the Supplementary material.
