# Supplementary material for: Autologous bone marrow mononuclear cell administration for neurological sequelae after traumatic brain injury: a matched control study
Source: Brain Commun. 2025 Sep 23;7(5):fcaf361. doi: 10.1093/braincomms/fcaf361 (PMC12501777; doi:10.1093/braincomms/fcaf361)
Supplement: fcaf361_Supplementary_Data [file fcaf361_supplementary_data.zip › Supplementary_Figure_Legends.docx]

**Supplementary Figure 1.** **Hematological parameters in the ABMMNC group at baseline and 6 months**

**(A)** Red blood cell count (RBC, T/L): 5.2 ± 0.6 at baseline, 5.2 ± 0.5 at 6 months (paired t-test, two-tailed, N = 25, p = 0.0995). **(B)** White blood cell count (WBC, G/L): 7.8 ± 2.2 at baseline, 7.8 ± 3.4 at 6 months (p = 0.9064). **(C)** Platelet count (PLT, G/L): 257.4 ± 60.2 at baseline, 244.8 ± 48.4 at 6 months (p = 0.2379). **(D)** Hemoglobin concentration (HGB, G/L): 149.2 ± 15.1 at baseline, 152.7 ± 14.8 at 6 months (p = 0.0159). Statistical analysis: Paired t-test, two-tailed, N = 25, comparing each parameter at 6 months with baseline within the group. Abbreviations: RBC, red blood cell count; WBC, white blood cell count; PLT, platelet count; HGB, hemoglobin concentration; ABMMNC, autologous bone marrow mononuclear cell; SD, standard deviation; T/L, tera per liter (10¹²/L); G/L, giga per liter (10⁹/L).

**Supplementary Figure 2. Coagulation parameters in the ABMMNC group at baseline and 6 months**

**(A**) Prothrombin time (PT, seconds): 13.3 ± 0.7 at baseline, 12.8 ± 0.9 at 6 months (paired t-test, two-tailed, N = 25, p = 0.0443). (**B**) Thrombin time (TT, seconds): 17.1 ± 1.3 at baseline, 16.1 ± 1.3 at 6 months (p = 0.0046). (**C**) Activated partial thromboplastin time (APTT, seconds): 33.1 ± 2.5 at baseline, 33.7 ± 1.7 at 6 months (p = 0.2395). Statistical analysis: Paired t-test, two-tailed, N = 25, comparing each parameter at 6 months with baseline within the group. Abbreviations: PT, prothrombin time; TT, thrombin time; APTT, activated partial thromboplastin time; ABMMNC, autologous bone marrow mononuclear cell; SD, standard deviation; s, seconds.

**Supplementary Figure 3. Liver and kidney function in the ABMMNC group at baseline and 6 months**

**(A**) Alanine aminotransferase (ALT, U/L): 33.1 ± 17.5 at baseline, 27.7 ± 16.7 at 6 months (paired t-test, two-tailed, N = 25, p = 0.1579). **(B**) Aspartate aminotransferase (AST, U/L): 21.3 ± 7.0 at baseline, 27.6 ± 19.1 at 6 months (paired t-test, two-tailed, N = 25, p = 0.093). **(C)** Urea (mmol/L): 4.3 ± 1.5 at baseline, 4.3 ± 1.2 at 6 months (paired t-test, two-tailed, N = 25, p = 0.9385). **(D)** Creatinine (µmol/L): 59.1 ± 10.8 at baseline, 59.7 ± 15.7 at 6 months (paired t-test, two-tailed, N = 25, p = 0.778). Statistical analysis: Paired t-test, two-tailed, N = 25, comparing each parameter at 6 months with baseline within the group.

Abbreviations: ALT, alanine aminotransferase; AST, aspartate aminotransferase; ABMMNC, autologous bone marrow mononuclear cell; SD, standard deviation; U/L, unit per liter; mmol/L, millimole per liter; µmol/L, micromole per liter.

**Supplementary Figure 4. Total FIM scores in the ABMMNC and control groups over 12 months**

**(A)** Mean total FIM scores (± SD) in the ABMMNC group at baseline, 3, 6, and 12 months (baseline: 55.1 ± 20.3; 3 months: 63.4 ± 22.5; 6 months: 66.2 ± 24.1; 12 months: 74.3 ± 24.1). **(B)** Mean total FIM scores (± SD) in the control group at the same time points (baseline: 51.2 ± 18.1; 3 months: 54.5 ± 19.4; 6 months: 58.7 ± 21.5; 12 months: 62.7 ± 23.8).

Within each group, paired t-tests (two-tailed, N = 25) were performed to compare each follow-up time point with baseline. All time points showed significant improvements compared to baseline (ABMMNC: 3 months, p < 0.0001; 6 months, p < 0.0001; 12 months, p < 0.0001. Control: 3 months, p = 0.0008; 6 months, p = 0.0001; 12 months, p = 0.0001).

Abbreviations: FIM, Functional Independence Measure; ABMMNC, autologous bone marrow mononuclear cell; SD, standard deviation.

**Supplementary Figure 5. Motor FIM scores in the ABMMNC and control groups over 12 months**

**(A)** Mean FIM motor scores (± SD) in the ABMMNC group at baseline, 3, 6, and 12 months. **(B)** Mean FIM motor scores (± SD) in the control group at the same time points.

In the ABMMNC group, scores increased from 37.2 ± 16.9 at baseline to 51.3 ± 20.1 at 12 months (paired t-test, N = 25, p < 0.0001). In the control group, scores increased from 34.0 ± 13.4 to 42.6 ± 18.8 (paired t-test, N = 25, p = 0.0002).

Abbreviations: FIM, Functional Independence Measure; ABMMNC, autologous bone marrow mononuclear cell; SD, standard deviation.

**Supplementary Figure 6. Cognitive FIM scores in the ABMMNC and control groups over 12 months**

**(A)** Mean FIM cognitive scores (± SD) in the ABMMNC group at baseline, 3, 6, and 12 months.

**(B)** Mean FIM cognitive scores (± SD) in the control group at the same time points.

In the ABMMNC group, scores increased from 18.0 ± 5.7 at baseline to 23.0 ± 5.2 at 12 months (paired t-test, N = 25, p < 0.0001). In the control group, scores increased from 17.2 ± 5.8 to 20.0 ± 6.0 (paired t-test, N = 25, p = 0.0006).

Abbreviations: FIM, Functional Independence Measure; ABMMNC, autologous bone marrow mononuclear cell; SD, standard deviation.

**Supplementary Figure 7. (A) PET-CT image of patient PID 05 at baseline. (B) PET-CT image at 12 months after ABMMNC therapy, showing increased FDG metabolism in the left occipital and temporal lobes.**

Statistical analysis: Qualitative imaging assessment; no quantitative statistical test was performed. Sample size: N = 9.

Abbreviations: FDG, fluorodeoxyglucose; PET-CT, positron emission tomography–computed tomography; ABMMNC, autologous bone marrow mononuclear cell.
