# Supplementary material for: Autologous bone marrow mononuclear cell administration for neurological sequelae after traumatic brain injury: a matched control study
Source: Brain Commun. 2025 Sep 23;7(5):fcaf361. doi: 10.1093/braincomms/fcaf361 (PMC12501777; doi:10.1093/braincomms/fcaf361)
Supplement: fcaf361_Supplementary_Data [file fcaf361_supplementary_data.zip › Supplementary_Tables 1-7.docx]

**Supplementary Table 1. Average characteristics of the use of ABMMNC infusion for treating neurological sequelae after TBI (first and second infusions)**

| **Parameter** | **N** | **First infusion** | | **Second infusion** | |
| --- | --- | --- | --- | --- | --- |
|  |  | **Mean ± SD** | **Median (Min–Max)** | **Mean ± SD** | **Median (Min–Max)** |
| MNC count/kg (×10⁶) | 25 | 19.0 ± 4.6 | 20.0 (12.0–29.2) | 16.2 ± 5.1 | 17.6 (7.8–28.8) |
| CD34+ cell count/kg (×10⁶) | 25 | 0.4 ± 0.2 | 0.4 (0.2–0.7) | 0.4 ± 0.2 | 0.4 (0.2–0.7) |
| MSC count/kg (×10⁶) | 25 | 0.004 ± 0.002 | 0.003 (0.0004–0.009) | 0.003 ± 0.002 | 0.003 (0.0004–0.009) |
| Cell viability (%) | 25 | 98.4 ± 1.3 | 98.7 (93.7–99.6) | 98.0 ± 1.4 | 98.4 (93.9–99.2 ) |

****ABMMNC****: Autologous Bone Marrow Mononuclear Cell;* ***TBI****: Traumatic brain injury;* ***MNC****: Mononuclear cell ;* ***MSC****: Mesenchymal stem cells*

**Supplementary Table 2. Summary of adverse events (AEs) and serious adverse events (SAE) by treatment group**

| **No.** | **AE/SAE description** | **SAE/AE** | **ABMMNC** | **Control** | **Relevance** |
| --- | --- | --- | --- | --- | --- |
| 1 | Mild infusion-site pain | AE | 3 | 0 | Related |
| 2 | Minor bleeding at the infusion site | AE | 1 | 0 | Related |
| 3 | Swelling at the bone marrow aspiration site | AE | 1 | 0 | Related |
| 4 | Swelling with discomfort in the right forearm | AE | 1 | 0 | Related |
| 5 | Hand numbness | AE | 1 | 0 | Related |
| 6 | Nausea | AE | 1 | 0 | Related |
| 7 | Dizziness | AE | 1 | 0 | Related |
| 8 | Myoclonic jerking/stridor | SAE | 1 | 0 | Not relevant |
| 9 | Respiratory failure and aspiration pneumonia | SAE | 1 | 0 | Not relevant |
| 10 | Headache | AE | 3 | 2 | Not relevant |
| 11 | Shoulder/arm pain or inflammation | AE | 2 | 0 | Not relevant |
| 12 | Seizures | AE | 1 | 2 | Not relevant |
| 13 | Mild fever | AE | 2 | 0 | Not relevant |
| 14 | Nausea | AE | 2 | 0 | Not relevant |
| 15 | Elevated liver enzymes | AE | 2 | 0 | Not relevant |
| 16 | Dermatitis/skin inflammation | AE | 3 | 0 | Not relevant |
| 17 | Muscle pain | AE | 3 | 1 | Not relevant |
| 18 | Menstrual disorder | AE | 1 | 0 | Not relevant |
| 19 | Chest pain | AE | 1 | 0 | Not relevant |
| 20 | Mild inflammation in the head region | AE | 1 | 0 | Not relevant |
| 21 | Leukocytosis | AE | 1 | 0 | Not relevant |
| 22 | Abdominal distension/ileus of unknown origin | AE | 1 | 0 | Not relevant |
| 23 | Diarrhea | AE | 1 | 0 | Not relevant |
| 24 | Acute exacerbation of chronic pyelonephritis, bilateral kidney stones, contrast agent allergy | AE | 1 | 0 | Not relevant |
| 25 | Ventricular premature beats | AE | 1 | 0 | Not relevant |
| 26 | Throat irritation | AE | 1 | 0 | Not relevant |
| 27 | COVID-19 infection | AE | 1 | 0 | Not relevant |
| 28 | Hyperglycemia | AE | 1 | 0 | Not relevant |
| 29 | Chickenpox | AE | 0 | 1 | Not relevant |
| 30 | Respiratory symptoms (nasal congestion and cough) | AE | 0 | 2 | Not relevant |
| 31 | Wound pain (weather-associated) | AE | 0 | 1 | Not relevant |

**Supplementary Table 3. Comparison of changes in motor FIM scores over time between the ABMMNC group and the control group via a mixed-effects model**

| **Model Parameter** | **Estimate ± SE** | **95% CI** | **p** |
| --- | --- | --- | --- |
| Intercept (Control group) | 34.0 ± 3.6 | [27.0; 41.0] | 0.529 |
| Baseline comparison | 3.2 ± 5.0 | [-6.7; 13.0] |  |
| Time point # Treatment group |  |  |  |
| 3 months # ABMMNC group | 4.8 ± 1.8 | [1.3; 8.4] | 0.008 |
| 6 months # ABMMNC group | 3.3 ± 1.8 | [-0.2; 6.9] | 0.067 |
| 12 months # ABMMNC group | 4.3 ± 1.8 | [0.7; 7.8] | 0.02 |

****ABMMNC****: Autologous Bone Marrow Mononuclear Cell; 'Intercept (Control group)' represents the baseline FIM score of the control group. 'Baseline comparison' indicates the estimated difference between ABMMNC group and the control group at baseline. 'Time point × treatment group' represents the change in FIM scores at each time point for the ABMMNC group compared to the control group.*

**Supplementary Table 4. Comparison of changes in cognitive FIM scores over time between the ABMMNC group and the control group via a mixed-effects model**

| **Model Parameter** | **Estimate ± SE** | **95% CI** | **p** |
| --- | --- | --- | --- |
| Intercept (Control group) | 17.2 ± 1.2 | [14.9; 19.5] | 0.643 |
| Baseline comparison | 0.8 ± 1.6 | [-2.4; 3.9] |  |
| Time point # Treatment group |  |  |  |
| 3 months # ABMMNC group | 0.3 ± 0.6 | [-1.0; 1.5] | 0.7 |
| 6 months # ABMMNC group | 0.2 ± 0.6 | [-1.1; 1.5] | 0.742 |
| 12 months # ABMMNC group | 1.4 ± 0.6 | [0.4; 3.0] | 0.009 |

****ABMMNC****: Autologous Bone Marrow Mononuclear Cell; 'Intercept (Control group)' represents the baseline FIM score of the control group. 'Baseline comparison' indicates the estimated difference between ABMMNC group and the control group at baseline. 'Time point × treatment group' represents the change in FIM scores at each time point for the ABMMNC group compared to the control group.*

**Supplementary Table 5. Improvement in SF-36 physical domain scores at each time point compared to baseline in each group**

| **Domains** | **Treatment** | **Baseline** | **3 months** | **p** | **6 months** | **p** | **12 months** | **p** |
| --- | --- | --- | --- | --- | --- | --- | --- | --- |
| Physical | ABMMNC group | 0 (0-10) | 15 (0-35) | 0.0002 | 25 (5-40) | 0.0000 | 37.5 (2.5-57.5) | 0.0000 |
|  | Control group | 0 (0-5) | 0 (0-5) | 1.0000 | 0 (0-15) | 0.0156 | 5 (0-20) | 0.0024 |
| Role limitations due to physical | ABMMNC group | 0 (0–0) | 0 (0–0) | 1.000 | 0 (0–0) | 1.000 | 0 (0–12.5) | 0.0313 |
|  | Control group | 0 (0–0) | 0 (0–0) | 1.000 | 0 (0–0) | 1.000 | 0 (0–0) | 1.000 |
| Pain | ABMMNC group | 67.5 (45–100) | 100 (80–100) | <0.001 | 100 (100–100) | <0.001 | 100 (100–100) | <0.001 |
|  | Control group | 57.5 (47.5–100) | 78.8 (55–100) | 0.023 | 83.8 (62.5–100) | 0.003 | 80 (55–100) | 0.006 |
| General health | ABMMNC group | 35 (35–45) | 50 (45–55) | <0.001 | 60 (50–65) | <0.001 | 60 (55–72.5) | <0.001 |
|  | Control group | 35 (30–45) | 45 (40–45) | <0.001 | 47.5 (45–50) | <0.001 | 47.5 (40–55) | <0.001 |

*** ***ABMMNC****: Autologous Bone Marrow Mononuclear Cell; Data are presented as median (range)*

Supplementary Table 6. Improvement in SF-36 mental domain scores at each time point compared to baseline in each group

| **Domains** | **Treatment** | **Baseline** | **3 months** | **p** | **6 months** | **p** | **12 months** | **p** |
| --- | --- | --- | --- | --- | --- | --- | --- | --- |
| Vitality | ABMMNC group | 50 (40–60) | 60 (50–70) | 0.002 | 65 (60–75) | <0.001 | 70 (62.5–77.5) | <0.001 |
|  | Control group | 40 (35–50) | 45 (30–55) | 0.459 | 47.5 (42.5–57.5) | 0.005 | 52.5 (40–60) | 0.014 |
| Social functioning | ABMMNC group | 25 (12.5–37.5) | 50 (37.5–62.5) | 0.005 | 62.5 (50–75) | <0.001 | 75 (62.5–87.5) | <0.001 |
|  | Control group | 37.5 (25–50) | 37.5 (31.25–56.25) | 0.02 | 50 (37.5–62.5) | 0.002 | 56.25 (31.25–81.25) | 0.004 |
| Role limitations due to emotional | ABMMNC group | 0 (0–0) | 0 (0–0) | 0.187 | 0 (0–0) | 0.083 | 83.33 (0–100) | 0.001 |
|  | Control group | 0 (0–0) | 0 (0–33.33) | 0.046 | 0 (0–0) | 0.187 | 0 (0–83.33) | 0.038 |
| Mental health | ABMMNC group | 52 (40–60) | 64 (60–76) | <0.001 | 70 (64–80) | <0.001 | 80 (70–80) | <0.001 |
|  | Control group | 60 (52–68) | 66 (58–72) | 0.036 | 62 (52–70) | 0.07 | 66 (56–74) | 0.021 |

*** ***ABMMNC****: Autologous Bone Marrow Mononuclear Cell; Data are presented as median (range)*

**Supplementary Table 7. Comparison of the improvement rates in GOSE classification between the two groups at 3, 6, and 12 months compared with baseline**

| **Time point** | **Status** | **ABMMNC**  **(n=25)** | **Control group (n=25)** | **Chi-square** | **p** |
| --- | --- | --- | --- | --- | --- |
| 3 months | No improvement | 25 (100%) | 22 (91.7%) | 2.172 | 0.141 |
|  | Improvement | 0 (0%) | 2 (8.3%) |  |  |
| 6 months | No improvement | 21 (84.0%) | 21 (87.5%) | 0.1225 | 0.726 |
|  | Improvement | 4 (16%) | 3 (12.5%) |  |  |
| 12 months | No improvement | 17 (73.9%) | 19 (79.2%) | 0.1808 | 0.671 |
|  | Improvement | 6 (26.1%) | 5 (20.8%) |  |  |

***ABMMNC****: Autologous Bone Marrow Mononuclear Cell;* ***GOSE****: Glasgow Outcome Scale–Extended*
