# Supplementary figures and images for: Autologous bone marrow mononuclear cell administration for neurological sequelae after traumatic brain injury: a matched control study
Source: Brain Commun. 2025 Sep 23;7(5):fcaf361. doi: 10.1093/braincomms/fcaf361 (PMC12501777; doi:10.1093/braincomms/fcaf361)

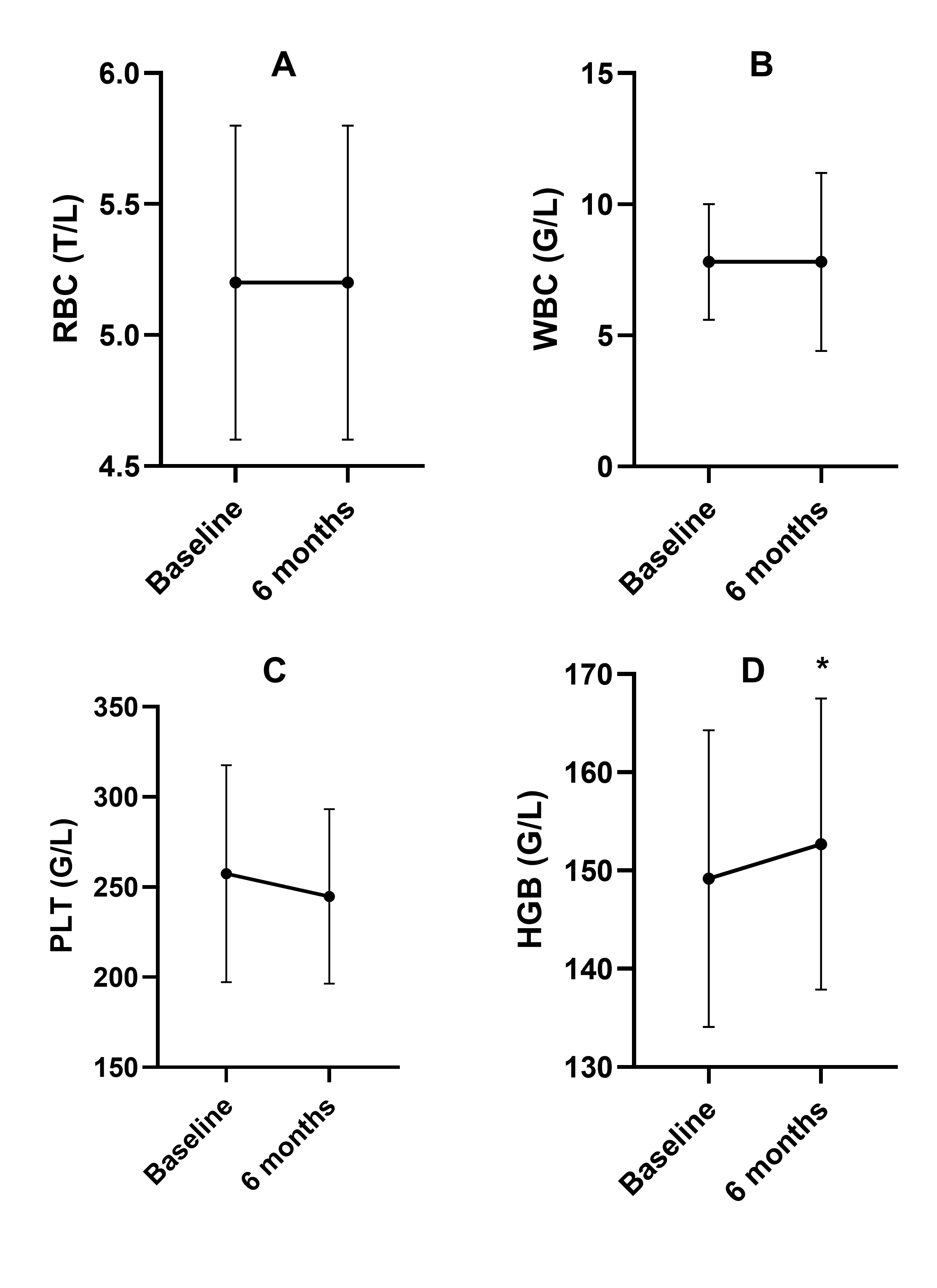

Supplement: fcaf361_Supplementary_Data [file fcaf361_supplementary_data.zip › Supplementary Figure 1.tif]

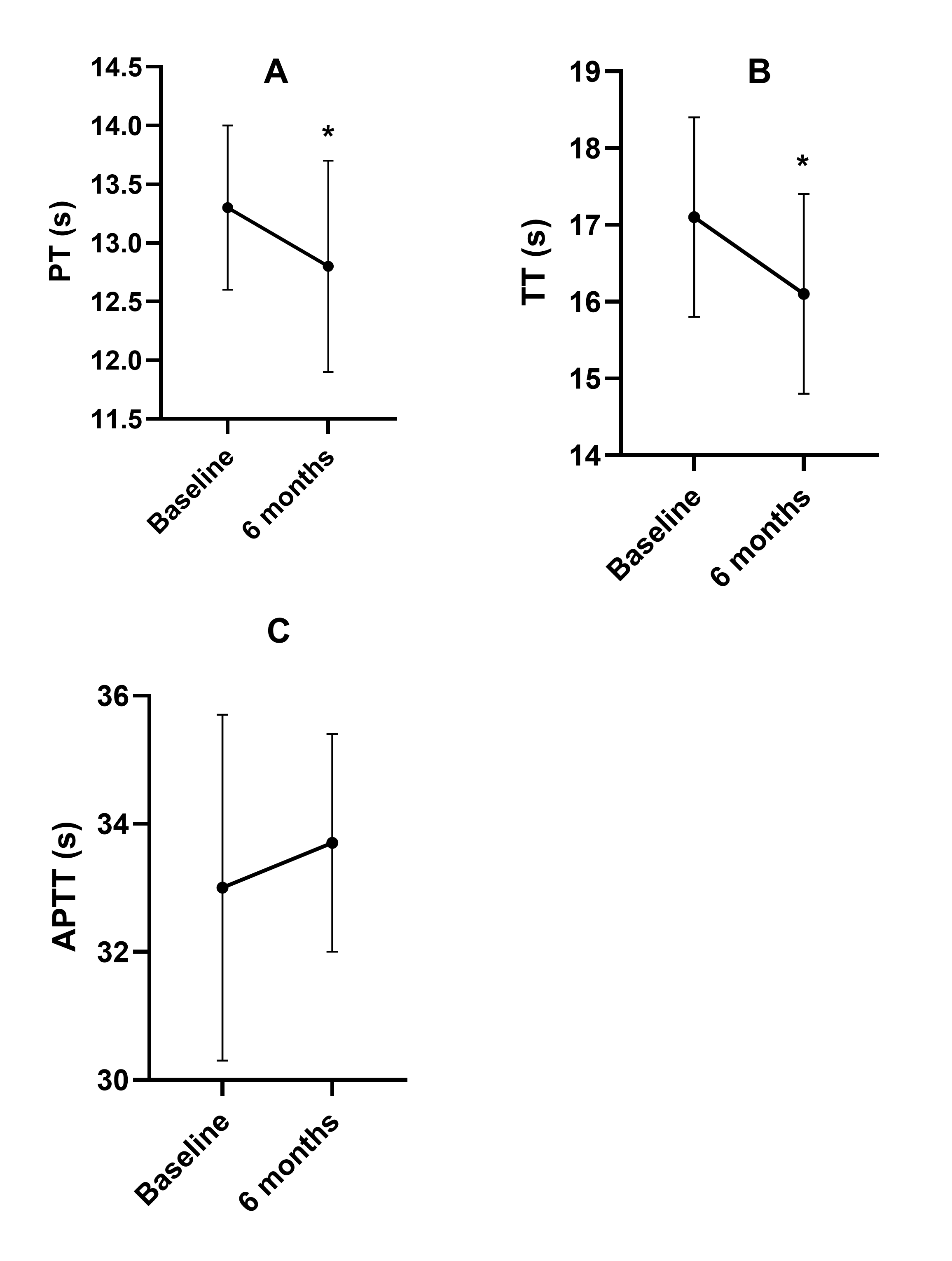

Supplement: fcaf361_Supplementary_Data [file fcaf361_supplementary_data.zip › Supplementary Figure 2.tif]

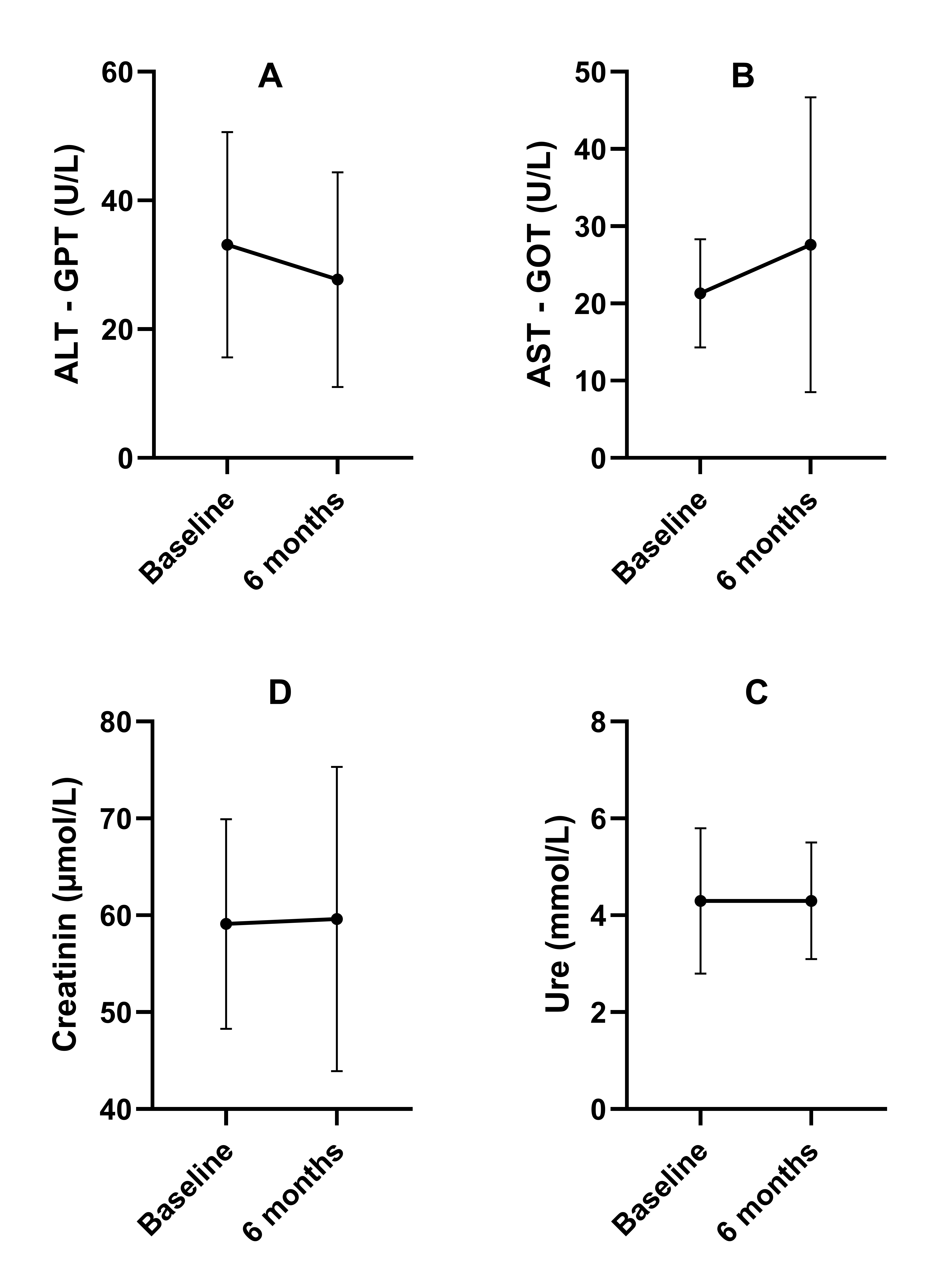

Supplement: fcaf361_Supplementary_Data [file fcaf361_supplementary_data.zip › Supplementary Figure 3.tif]

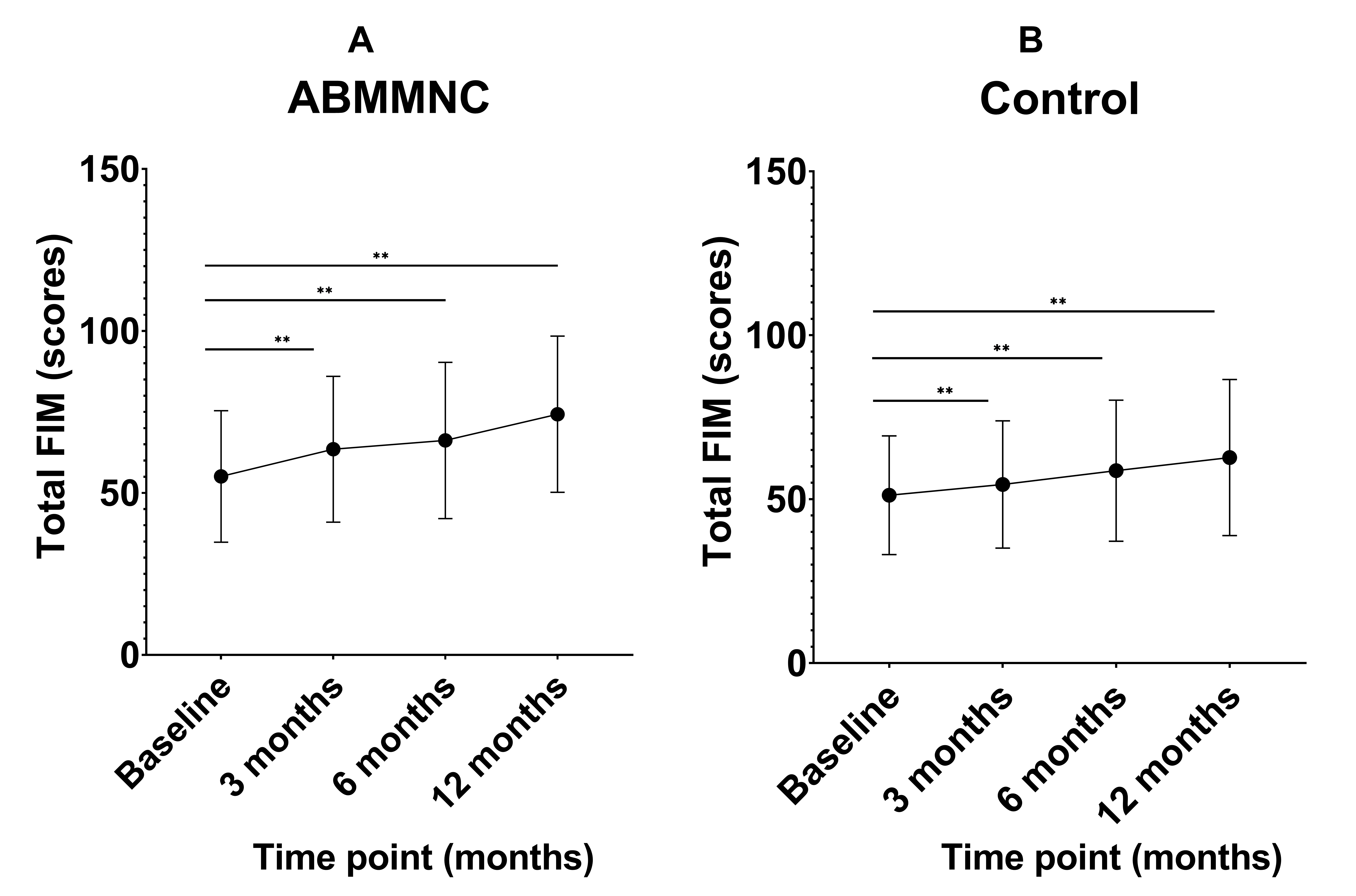

Supplement: fcaf361_Supplementary_Data [file fcaf361_supplementary_data.zip › Supplementary Figure 4.tif]

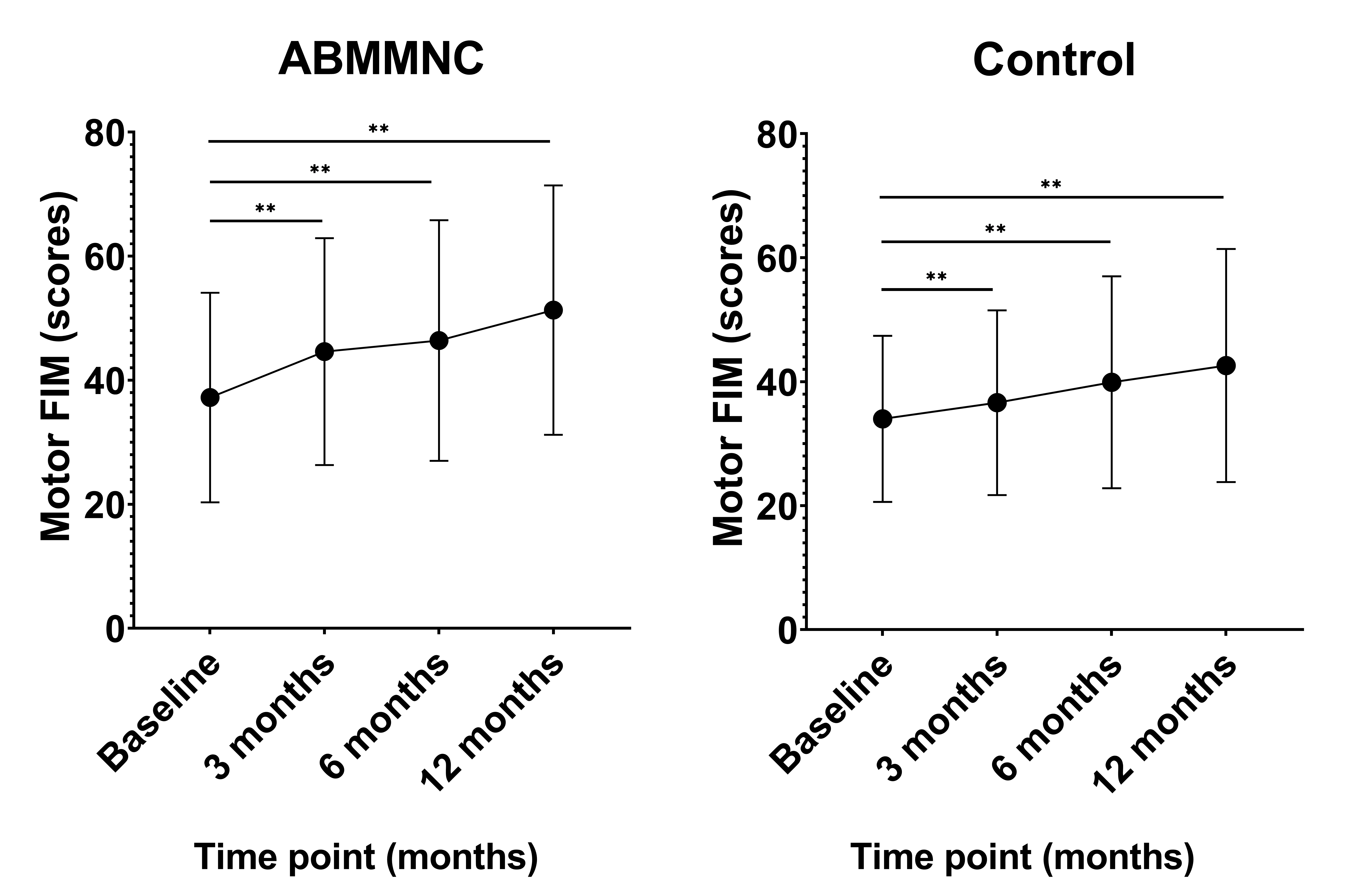

Supplement: fcaf361_Supplementary_Data [file fcaf361_supplementary_data.zip › Supplementary Figure 5.tif]

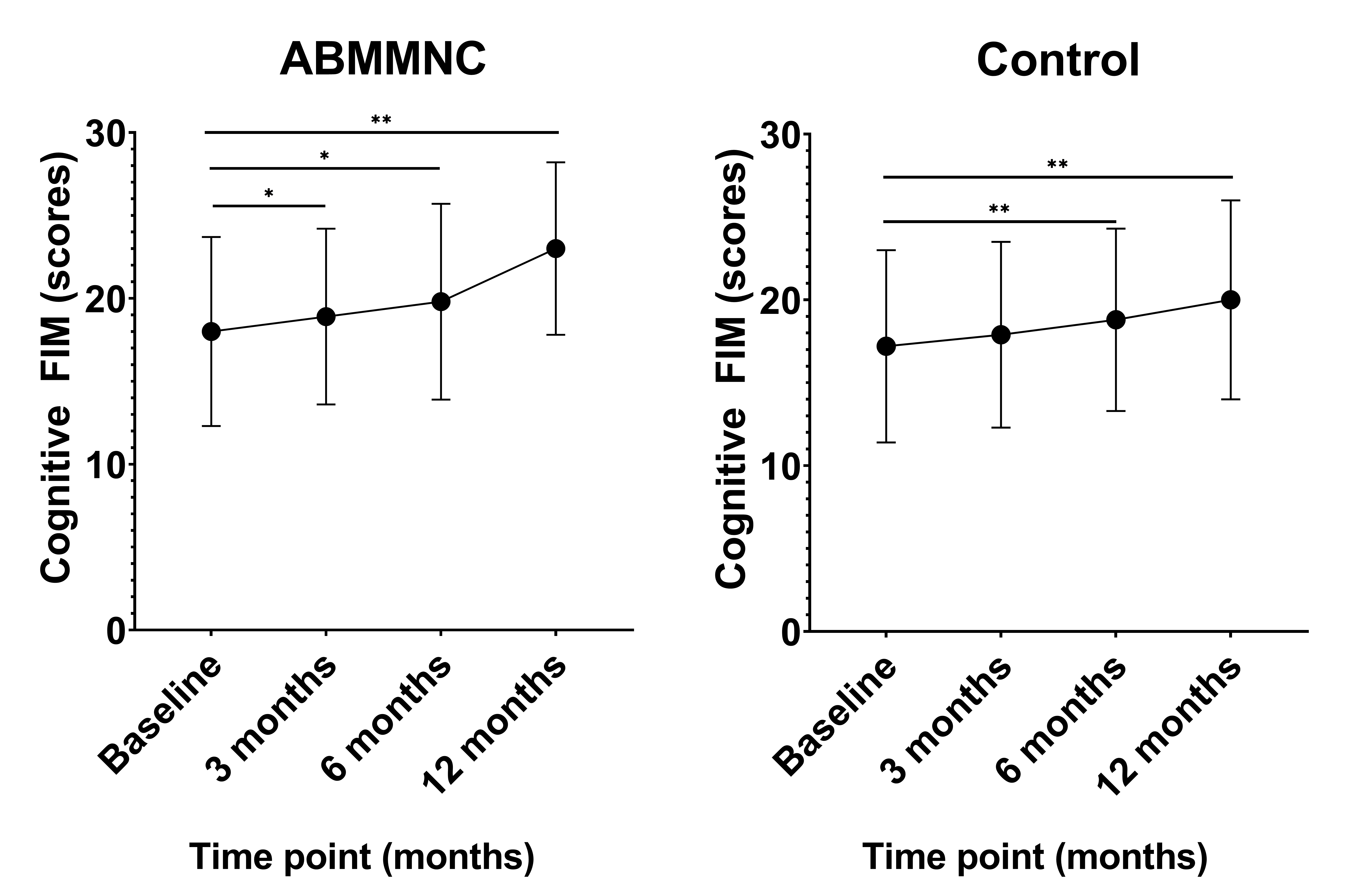

Supplement: fcaf361_Supplementary_Data [file fcaf361_supplementary_data.zip › Supplementary Figure 6.tif]

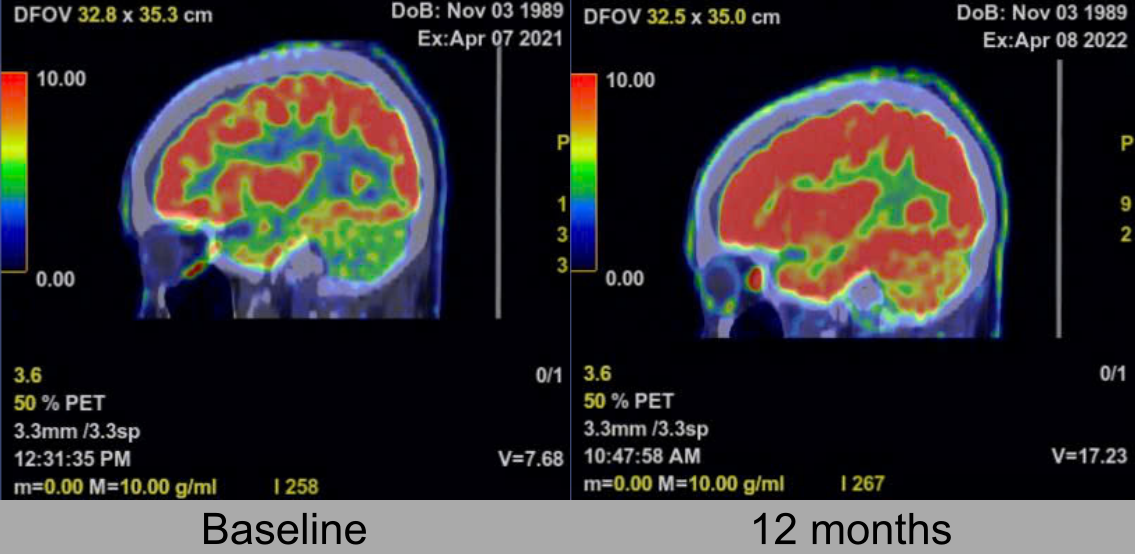

Supplement: fcaf361_Supplementary_Data [file fcaf361_supplementary_data.zip › Supplementary Figure 7.tiff]
